# Upadacitinib Treatment for Fulminant Cutaneous and Mucosal Lichen Planus

**DOI:** 10.7759/cureus.108423

**Published:** 2026-05-07

**Authors:** Aarushi Gulati, Brittany P Smirnov

**Affiliations:** 1 Dermatology, A.T. Still University, Mesa, USA; 2 Dermatology, Fuchs Dermatology, Fairfax, USA

**Keywords:** fulminant lichen planus, lichen planus, mucosal lichen planus, oral lichen planus, upadacitinib

## Abstract

We report an otherwise healthy 24-year-old woman with a fulminant presentation of cutaneous and mucosal lichen planus (LP), progressing from approximately 15% to 80% total body surface area (BSA) in four weeks without an identifiable drug or infectious trigger. Oral examination was initially negative for signs of mucosal LP, such as Wickham striae and associated erosions. Subtle Wickham striae developed at the labial frenulum with mild sensitivity to spicy foods by week four. Laboratory evaluation, including complete blood count (CBC), comprehensive metabolic panel, antinuclear antibody, hepatitis panel, and human immunodeficiency virus testing, was unremarkable. Punch biopsy demonstrated hyperkeratosis, hypergranulosis, irregular acanthosis, and a vacuolar lichenoid interface dermatitis consistent with LP. The patient began treatment with clobetasol 0.05% cream applied to the affected areas of the wrists, elbows, knees, and feet as well as triamcinalone 0.1% cream applied to the affected areas of the back, chest, arms, and legs. Both medications were applied twice daily as needed for two weeks per month for one month total. As the patient was refractory to this treatment, the patient was treated with tacrolimus 0.1% ointment and ruxolitinib 1.5% cream twice daily for four weeks without adequate improvement, still reporting 10/10 pruritus. Disease was refractory to topical corticosteroids and subsequent topical anti-inflammatory therapy. Subsequently, she was started on systemic treatment with oral upadacitinib at a dose of 15 mg daily. Systemic treatment with upadacitinib led to rapid improvement with resolution of pruritus and regression of plaques within three weeks. After three weeks, plaques regressed and pruritus resolved (0/10). The patient described increased quality of life as a result of treatment.

This case depicts a severe, rapidly progressive LP in a younger adult without comorbidities or risk factors. Similar presentations warrant early clinicopathologic confirmation and timely therapeutic escalation, including systemic options such as upadacitinib, to limit symptom progression and complications of extensive disease.

## Introduction

In the current literature, lichen planus (LP) is described as a chronic T cell-mediated inflammatory condition with mucocutaneous manifestations [1,2]. Characteristic findings include purple pruritic polygonal papules and mucosal epidermal hyperkeratosis known as Wickham striae [1,3]. Predisposing factors include genetic susceptibility and infectious or environmental triggers such as koebnerization, psychological stress, and drug exposures [1,4]. LP typically progresses gradually over weeks to months and most commonly presents in middle-aged adults between 30 and 60 years of age [5]. LP is classified as a lichenoid interface dermatitis characterized histopathologically by a band like lymphocytic infiltrate at the dermoepidermal junction with basal vacuolar degeneration and hypergranulosis [3,6]. Diagnosis is based on clinical findings supported by histopathology [6,7]. Standard management consists of high potency topical corticosteroids and topical calcineurin inhibitors and escalates to systemic immunomodulatory therapy for severe or refractory disease [5,7]. Treatment strategies may be modified depending on disease extent and severity, and emerging systemic immune modulatory approaches are currently being explored [2,8]. In exploring this evolving therapeutic backdrop, the management steps of fulminant LP are less clearly defined.

In this case report, we describe a fulminant presentation with rapid progression of cutaneous body surface area (BSA) involvement and new oral involvement in a young adult within a four week interval. While the clinical and histopathologic features of LP are well described, the literature less clearly defines management strategies for rapidly progressive, extensive cutaneous disease with emerging mucosal involvement in young adults [1,5-7]. This case presentation highlights atypical LP progression over a four week interval and explores a distinct therapeutic approach for extensive disease using systemic immune modulation with oral upadacitinib.

## Case presentation

**Figure 1:**
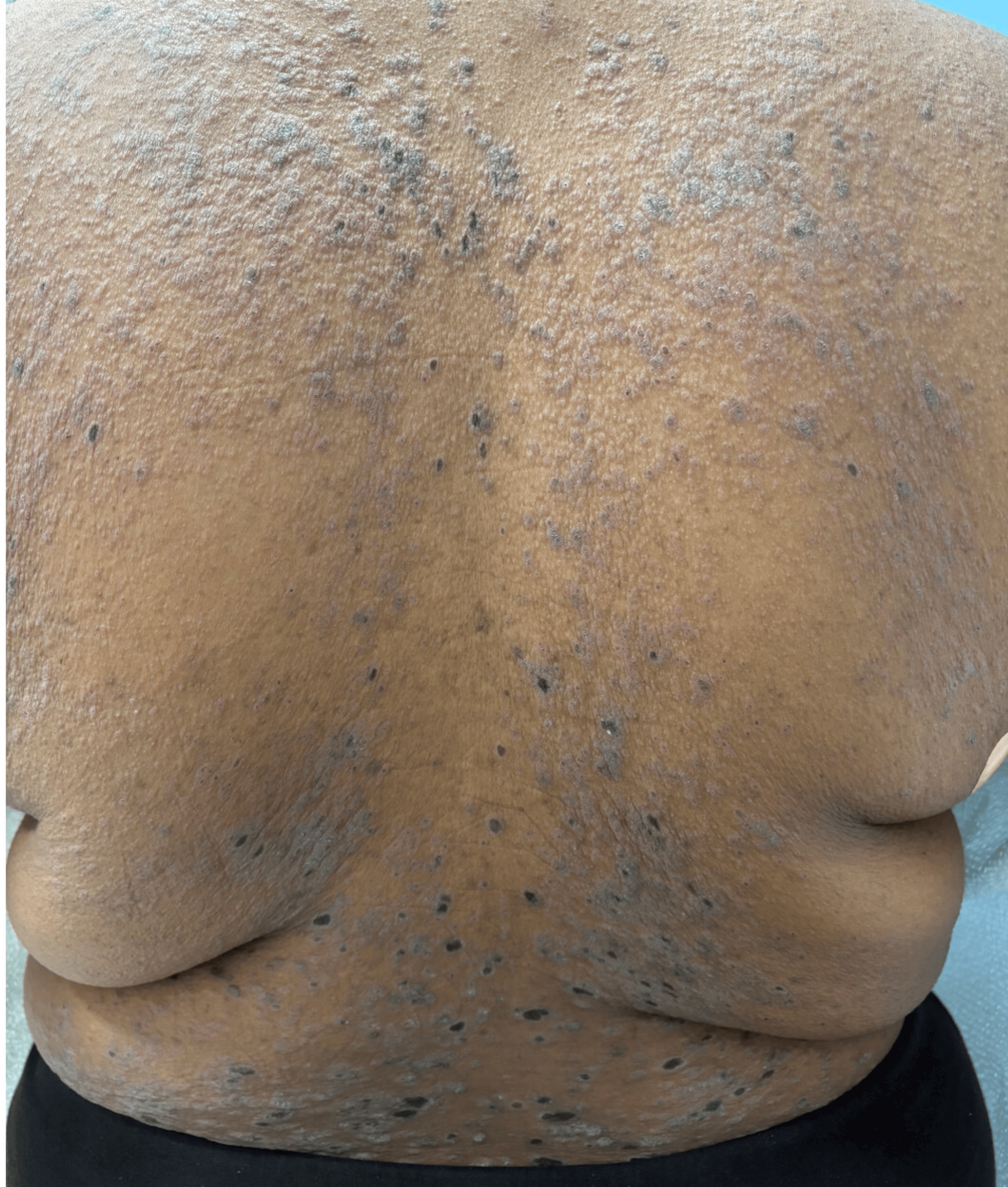
Diffuse violaceous papules and plaques on the posterior trunk, representing rapid progression of cutaneous disease to greater than 80% BSA over a four-week period BSA: Body surface area

**Figure 2:**
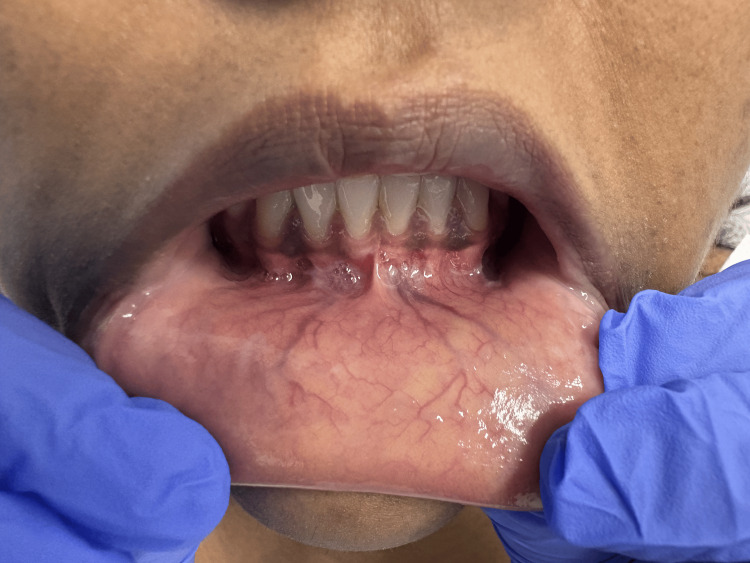
Oral manifestations of mucosal LP, including Wickham's striae at the base of the marginal gingiva LP: Lichen planus

A 24-year-old Indian American female patient with no chronic medical conditions and Fitzpatrick skin type V presented with a one-month history of rapidly progressive cutaneous disease. Cutaneous involvement increased from approximately 15% to 80% total BSA in four weeks. Skin changes were associated with 10/10 pruritus without concurrent pain and neuropathy. Examination demonstrated widespread hypertrophic violaceous papules and plaques with scale involving the trunk and extremities (Figure 1). Mucosal examination was initially negative; within four weeks, subtle Wickham striae developed at the labial frenulum with mild sensitivity to spicy foods (Figure 2). Nail involvement was absent.

**Figure 3:**
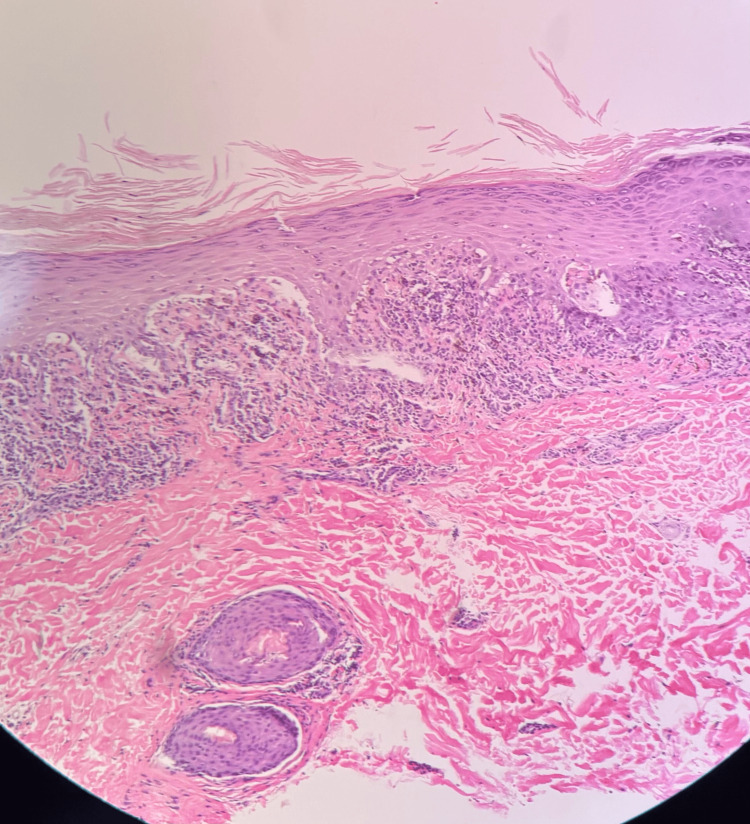
Cutaneous LP at 4x. Hematoxylin and eosin-stained section showing a lichenoid interface dermatitis with a dense band-like lymphocytic infiltrate at the dermoepidermal junction. LP: Lichen planus

**Figure 4:**
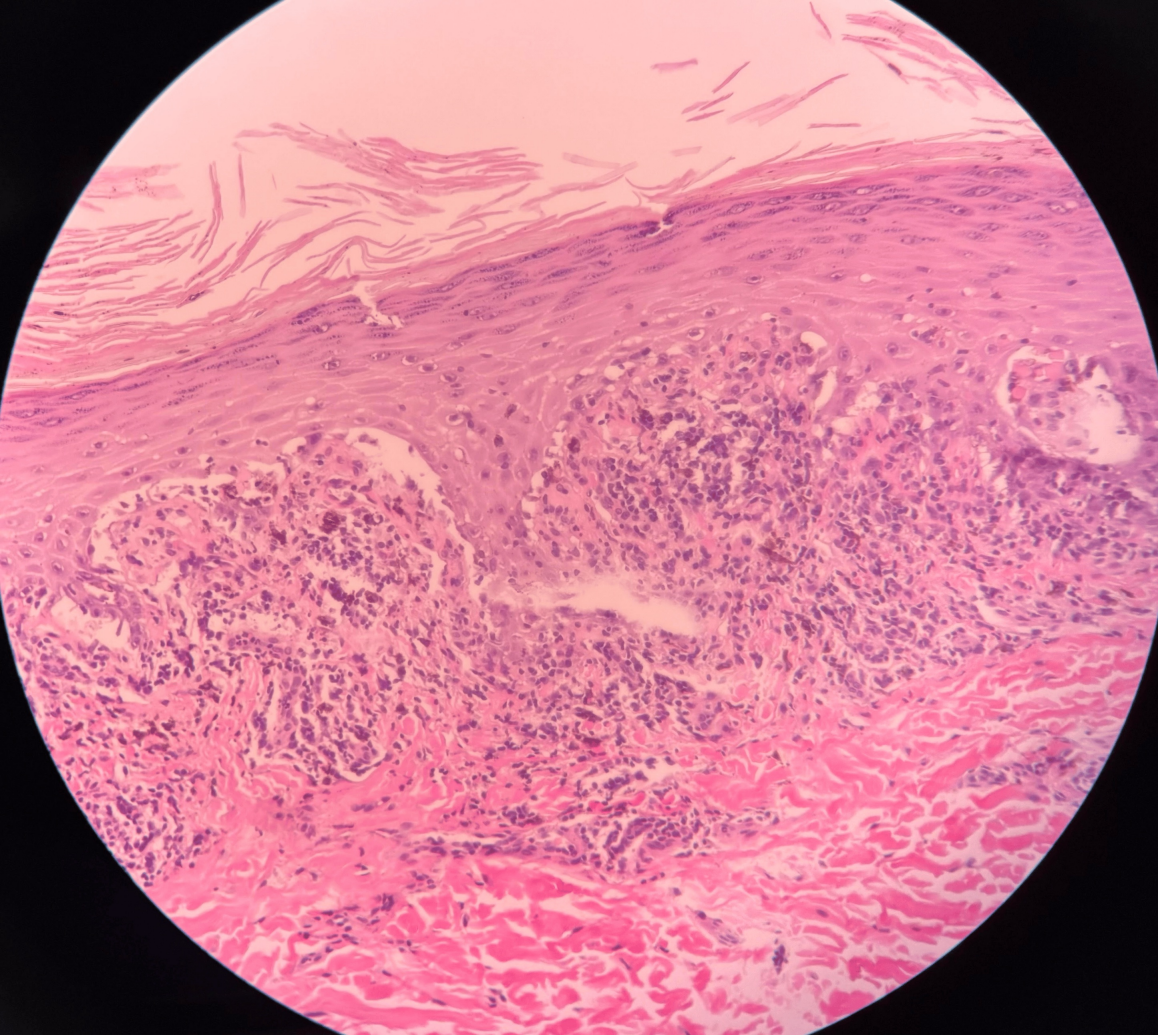
Cutaneous LP at 40x. Hematoxylin and eosin-stained section showing a dense band-like lymphocytic infiltrate closely apposed to the epidermis with basal layer injury, consistent with lichenoid interface dermatitis. LP: Lichen planus

**Figure 5:**
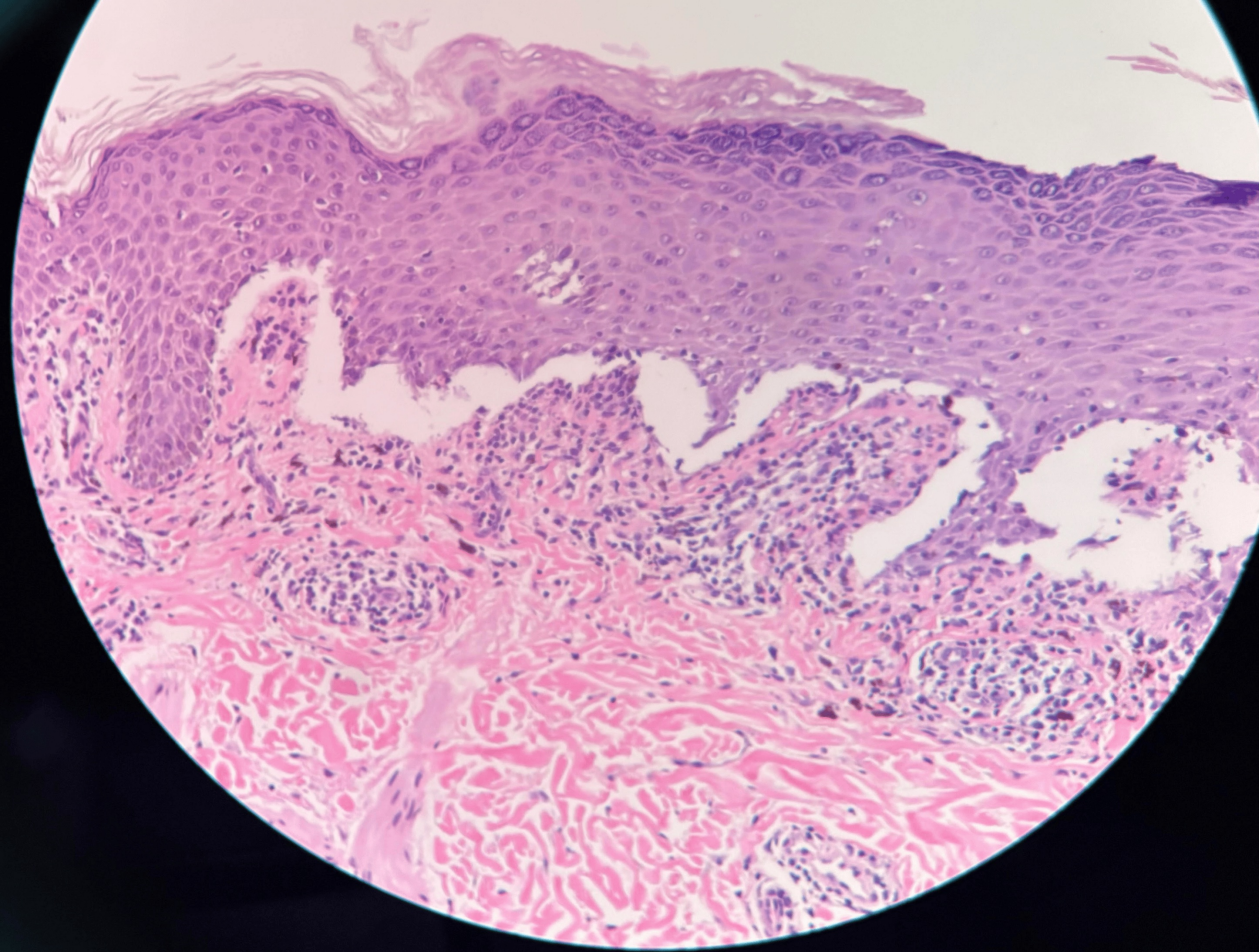
Oral LP at 40x (high power). Hematoxylin and eosin-stained mucosal section showing interface mucositis with irregular epithelial hyperplasia and a dense subepithelial lymphocytic infiltrate, compatible with oral LP. LP: Lichen planus

Complete blood count (CBC), comprehensive metabolic panel, antinuclear antibody, hepatitis panel, and human immunodeficiency virus testing were all within accepted ranges, which did not further elucidate any triggers, such as acute viral infection or autoimmunity, for the fulminant presentation. A 4 mm punch biopsy from the back showed hyperkeratosis, hypergranulosis, irregular jagged epidermal hyperplasia, and vacuolar lichenoid interface dermatitis consistent with LP (Figures 3, 4, 5).

The patient began treatment with clobetasol 0.05% cream applied to the affected areas of the wrists, elbows, knees, and feet as well as triamcinalone 0.1% cream applied to the affected areas of the back, chest, arms, and legs. Both medications were applied twice daily as needed for two weeks per month for one month total. Given continued progression of cutaneous involvement and persistent 10/10 pruritus despite two weeks of daily high potency topical corticosteroid therapy, the disease was considered insufficiently responsive to initial topical treatment. Thus, the patient was transitioned to topical tacrolimus 0.1% ointment and ruxolitinib 1.5% cream twice daily for four weeks; however, she continued to report severe 10/10 pruritus without clinical improvement in involved BSA. Subsequently, she was started on systemic treatment with oral upadacitinib at a dose of 15 mg daily. After three weeks, plaques regressed and pruritus resolved (0/10).

## Discussion

The majority of LP cases improve within one to two years, with oral LP often demonstrating a slower or incomplete resolution in refractory disease [5,7]. In contrast, this case demonstrates rapid progression to 65% total BSA involvement with new mucosal disease within four weeks. In rapidly progressive or widespread eruptions of LP, early biopsy is valuable to exclude infectious or drug induced processes that may mimic lichenoid disease [2,3]. This fulminant pattern broadens the clinical frame beyond the standard teaching and reinforces that LP should remain in the differential diagnosis even when progression occurs more rapidly than expected in patients that do not have known risk factors.

In this case, the extensive disease burden and severe pruritus refractory to topical therapy necessitated treatment escalation to prevent ongoing inflammatory injury and long term pigmentary change, which is particularly relevant in patients with higher Fitzpatrick skin types.

Immune dysregulation in LP has been associated with interferon signaling and downstream activation of the JAK STAT pathway [6]. Upadacitinib is an oral selective JAK1 inhibitor [8]. Although LP is not currently an approved indication, emerging case series and reports describe noteworthy clinical responses in refractory LP phenotypes treated with JAK inhibition [8], supporting a immunologically plausible therapeutic approach in patients with high disease burden. In this patient, both pruritus and cutaneous manifestations improved within three weeks of systemic upadacitinib therapy, demonstrating rapid clinical improvement after inadequate response to topical agents. Further investigation is needed to better characterize predictors of accelerated disease progression in LP, determine whether specific immunologic subtypes are more likely to exhibit fulminant presentations, and clarify the efficacy, durability, and safety of JAK inhibition across both cutaneous and mucosal LP phenotypes [2,6].

This case should be interpreted within the limitations of a single-patient observation. Although the temporal association between systemic upadacitinib initiation and clinical improvement was notable, spontaneous improvement, delayed response to prior topical therapy, or the effect of multimodal treatment cannot be fully excluded. Additionally, while emerging reports have described responses to JAK inhibition in refractory LP, the current literature remains limited to small case reports and case series rather than controlled studies [6,8]. Larger controlled studies evaluating novel systemic therapies will be essential to clarify efficacy, durability, and safety across both cutaneous and mucosal LP phenotypes [8]. Compared with the gradual clinical course typically described in LP, this case is notable for the rapid expansion of cutaneous involvement, new mucosal findings, severe pruritus, and urgent need for systemic escalation.

## Conclusions

Fulminant cutaneous and mucosal LP can present in otherwise healthy young adults without clear exposures to infections and environmental triggers. Early clinicopathologic confirmation and timely escalation to systemic therapy are critical considerations in extensive disease to reduce symptom burden and limit post-inflammatory hyperpigmentation. This case introduces a unique fulminant presentation of LP as well as efficacious management of the patient with systemic JAK inhibition. 
